# Our New York Letters

**Published:** 1892-02

**Authors:** Wm. L. Russell

**Affiliations:** 151 East 50th Street; New York


### Correspondence.

### OUR NEW YORK LETTERS.

NEW YORK, December 14th, 1891.

### UTERINE DRAINAGE FOR CHRONIC METRITIS AND ENDOMETRITIS.

This was the subject discussed at the last meeting of the Academy, where Prof. Wm. M. Polk read a paper in which he advocated a plan which had proved very successful in his hands. He stated that he believed that the timidity with which most writers regarded any interference with the interior of the uterus was misplaced. His experience with vaginal hysterectomy had taught him that if scrupulous cleanliness were observed, the cavity of the uterus could be entered with impunity. He had therefore been led to try the effect of packing with gauze in metritis and endometritis with and without salpingitis. In cases of puerperal or gonorrhœal origin this treatment was always pursued ; it had also been successful in a number due to flexions and other causes. In hemorrhagic cases curetting was first employed, followed by packing. The common dictum to avoid invading the uterus when the appendages or surrounding tissues were inflamed had not been respected by him, as he had resorted to packing in such cases with the hope of preventing the extension of disease or of modifying the conditions already existing. Rest, laxatives, hot douches and glycerine tampons were first tried ; then, if the disease persisted drainage was resorted to. If the procedure was properly conducted, he did not regard it as dangerous; if not, however, it might become so. He had treated forty cases, and the usual effect had been a rise of temperature during the first twenty-four or forty-eight hours, to be followed by a fall which was permanent.

His mode of operating was as follows: The interior of the uterus was washed out with a solution of bichloride 1 to 5,000. In puerperal cases the os was usually sufficiently patulous. In other cases it would have to be dilated. In such instances the vulva, vagina and cervical canal were cleansed as for a hysterectomy. Dilators were then introduced into the cervical canal until it became sufficiently enlarged to admit a cervical speculum. As this was a rather painful procedure an anæsthetic was usually required. After the uterine cavity had been irrigated, curetting was done if necessary. While the preceding steps were being taken, strips of sterilized cheese cloth, three feet long and three-quarters of an inch wide should have been soaking in a bichloride solution of 1 to 500. These should now be removed and thrown into hot water. From this they were taken and transferred to the cavity of the uterus which was completely filled. This was accomplished by means of a screw which he had devised, which could be more readily disengaged from the meshes of the gauze than any other instrument. The loose ends were brought out through the os, and after the speculum had been removed, were curled up against the cervix. A plug of the gauze was then placed in the vagina. If much pain followed the operation, hot fomentations to the abdomen ususally relieved it. At the end of six or seven days the plug should be removed, if uterine contractions had not already expelled it, and the patient might be allowed to rise. The longest duration recorded by him was two weeks. The amount of depletion from this plan of treatment was apparent in the profuse discharge observed on the second, third and fourth days.

Dr. W. T. Lusk said that he had had no experience with the plan of treatment suggested, but thought the principle was good, as no inflamed mucous membrane could recover while bathed in morbid secretions. He had employed similar measures in puerperal septic conditions, when the patient seemed moribund, sometimes with marvellous results. He believed that many tube inflammations originated in the cervix.

Dr. C. C. Lee stated that uterine drainage had been employed by him in a number of cases, but his method had been different from that pursued by Dr. Polk. He never used bichloride solutions within the uterus, as he had seen poisonous symptoms produced by this procedure. He used simple hot boiled water instead. His plan consisted in dilating the cervix with Palmer's or Dudley's dilators, then curetting and irrigating, and afterwards stitching into position one of Cleveland's perforated glass drainage tubes. This was kept in place for ten days, and was then replaced by a stem pessary, which was worn for a considerable period, the patient returning occasionally for observation.

Dr. W. G. Wylie thought that the introduction of the cervical speculum was impracticable, when there was stenosis of the os, and might result in splitting of the uterus.

Dr. Edebohls had seen a few cases in which diseased tubes had seemed to drain naturally through the uterus; in such cases he believed that packing would be very useful.

Dr. Thomas Addis Emmett said that he had formerly tried several methods of uterine drainage, in cases of subinvolution and endometritis, and had met with some success. The cures had not been permanent, however, and he abandoned the treatment. He had resorted to it again during the past two years, when distended tubes drained naturally into the uterus. As for metritis, he hardly knew what the term meant, as there was no true mucous membrane above the internal os. A new growth, or some condition due to injury or to parturition might demand treatment of the interior of the uterus. Where there was nothing but hypersecretion, the real disease must be looked for outside of the uterus. He believed, however, that drainage would prove useful for the treatment of diseased tubes.

The plan of treatment suggested by Dr. Polk seemed to meet with general approbation, and most of the speakers expressed their intention of giving it a trial.

### THE TREATMENT OF BUBOES.

A rather novel method of treatment of this troublesome condition was discussed at the last meeting of the Surgical Society. Dr. McBurney read a paper. He said that the old treatment was to paint with iodine; if suppuration seemed inevitable poulticing was resorted to, and when fluctuation was detected an incision was made. Five years ago he had adopted radical excision as a general rule of treatment. Now he divided his cases into three classes; those in which the glands were tender and hard without any sign of septic infection; those in which there were signs of gland deterioration, tenderness, redness, and softening; those in which the glands were completely broken down. Cases of the first class were treated by the application of cold. Those of the second were suitable for excision, all the glands being removed, and those portions of the skin which were badly involved in the inflammatory process. This treatment succeeded in proportion to the freedom of the glands from softening. It was in the third class of cases, in many of which excision was hopeless, that he had been led to adopt injections of iodoform and vaseline, as advised by Pontain. He had treated twenty cases. The method pursued was to make a small opening, through which the fluid was expressed. A small spoon was then introduced and dividing septa and connective tissue bands broken down. The sac was then overdistended six to twelve times with a bichloride solution 1 to 1000. Then by means of a syringe a mixture of iodoform and vaseline, a drachm to the ounce, which had been heated to fluidity, was injected. The application of a cold gauze compress caused the vaseline to harden in a few minutes. In two cases treated by him complete failure had resulted; in eight a partial success was secured, and in the remaining ten the success was complete after the first or second injection. Five had recovered before the fourteenth day, and the average duration of all was nineteen days.

Dr. Briddon said that he had wounded the saphena vein in operating for the excision of buboes, and was obliged to ligate it.

Dr. Pilcher, of Brooklyn, remarked that up to this time he had regarded the treatment by vaseline injection with scepticism. It seemed to him now that it might, perhaps, be best to let buboes mature, and then treat them in this way rather than to employ excision. Gangrene of the scrotum had followed one of his operations, probably from interference with innervation.

Dr. L. A. Stimson believed that the danger of wounding the large veins could be avoided if the operator made the discovery of their location the first step in the operation after the preliminary incision.

Dr. Groger said that since he had begun to treat chancroids by powdering the surface thickly with salicylic acid, buboes had seldom occurred.

Dr. Abbe had found that suppuration usually resulted after operation for excision. He had, therefore, abandoned suturing and packed the wound with gauze for thirty-six hours, when the granulating surfaces were allowed to fall together to heal by secondary adhesion.

In closing the discussion Dr. McBurney said that with the improved methods of wound treatment sutures could be used with very satisfactory results. Fifty per cent. of his cases healed by first intention. He thought that it was unnecessary to wait until a bubo became a pus sac before resorting to the vaseline injections.

He exhibited a case in which treatment had been begun four weeks before. It had been a very bad case, a tense sac of pus six inches across having existed. One injection only was employed. The place was now almost entirely healed, with the exception of a small sinus perfectly sweet and free from pus, but from which vaseline could be expressed.

### TREATMENT OF PULMONARY ŒDEMA.

An interesting discussion on this subject took place at the last meeting of the County Society. Dr. A. A. Smith read a paper. He stated that the cause of many cases of acute œdema of the lungs was not always apparent. Interference with the nervous supply was often the only satisfactory explanation, this being perhaps brought about by the poisonous effect of uræmia on the ganglionic system, causing it to lose its hold on the pulmonary blood vessels. Although a sudden attack of œdema of the lungs might be the first suggestion of kidney disease, such cases were rare, compared with those in which the condition was of gradual onset. Even when there was marked diminution in the quantity of urine excreted, pulmonary œdema did not always appear until the kidney disease had existed long enough to cause changes in the blood and in the walls of the vessels. A remarkable instance was that in which a kidney was removed from a patient in Bellevue Hospital, and no urine was excreted for eleven days, when the patient died. No pulmonary œdema appeared in this case, and yet the autopsy revealed that the woman's only kidney had been removed.

He had found the treatment by dry cupping unsatisfactory, and had therefore resorted to other measures. His custom was to administer strychnine and atropia hypodermically and nitroglycerine by the mouth. Atropia was a cardiac and respiratory stimulant; strychnine was also, and a vaso-motor stimulant as well. Nitroglycerine distributed the blood through the arterial system. He gave it in one one-hundredth of a grain dose every two hours. One-fiftieth of a grain of strychnine and one one-hundredth of a grain of atropia might be given every two hours for two doses, and afterwards every four hours. If renal disease were the cause of œdema, morphine was of the utmost value. If cyanosis were pronounced, oxygen was useful, care being taken not to put the tube too near the mouth.

Dr. Roosevelt said that he had not found the treatment by dry cupping entirely unsatisfactory. He had also used strychnine, in alcoholic cases to whom he had given one-thirtieth of a grain every half hour for four doses, the patient being watched carefully in the meantime for poisonous symptoms.

Dr. Kinnicutt had used nitroglycerine in one-fiftieth of a grain doses or smaller, being guided by the feelings of the patient in regard to flushing of the face, etc. His custom was to give the remedy up to the point of producing subjective symptoms, and he thought that it might be given every twenty minutes indefinitely or until the pulse grew soft, as its effects were so evanescent.

Dr. Andrew H. Smith said that sodium nitrate in two grain doses produced an effect similar to that of nitroglycerine, and much more lasting. He believed in the efficacy of opium in uræmic cases. Digitalis was positively contraindicated; its effect was to empty the arteries into the veins, while our object was to empty the veins into the arteries.

### TREATMENT OF PERITYPHLITIC ABSCESS.

The so-called simple incision in perityphlitic abscess was also discussed at the same meeting as the preceding subject. Dr. McBurney introduced the subject. He said that so long as it was considered that the abscess originated in the connective tissue outside the peritoneum it had been thought good treatment to wait until pointing occurred, before making any incision. Now that the part played by the appendix vermiformis was known, and the fact that pus did not appear outside the peritoneum until a late stage of the disease, other treatment had been instituted. It was not best to wait until the abscess reached the surface; the sac should be searched for and then opened carefully with a small incision to prevent flooding of the peritoneum. The position occupied by the appendix was uncertain, and the abscess might point in any direction. Most surgeons therefore operated early.

Dr. Gerster said that a case had come under his observation in which an aspirating needle had been used to detect pus. On this a grooved director was passed, and a free incision made. The result proved that the intestine had been incised, and a fæcal fistula resulted.

Dr. L. A. Stimson remarked that while the surgeon was waiting for the proper time to make the simple incision, the patient might die, or grow so bad as to render the operation useless.

There is some difference of opinion among the surgeons here regarding the propriety of an exploratory puncture by a fine needle to determine the existence of pus in these cases of perityphlitis. Dr. McBurney has stated that he has observed a case in which such a puncture would have penetrated the intestine four times. The case referred to by Dr. Gerster is also suggestive. On the other hand, there are some who, arguing from practical experience, say that they have used the needle in many cases and have never seen a bad result.

WM. L. RUSSELL.

151 *East 50th Street.*

NEW YORK, January 12, 1892.

A most interesting and important paper was read at the last meeting of the Academy by Dr. Virgil P. Gibney. The subject was

TUBERCULAR OSTEITIS OF THE KNEE.

The object of the paper, as stated by the reader, was not to present anything new, but rather to illustrate the methods of treatment at present pursued, and what results it was possible to secure. A large number of cases were exhibited whose movable joints and straight limbs spoke more forcibly than anything that could be said by the speaker.

The occasion was honored by the presence of the veteran orthopedic surgeon, Dr. L. A. Sayre. Dr. Sayre is a victim of chronic rheumatism or gout, and is now seldom seen at public meetings. His firm voice and vigorous manner of speaking, however, showed few signs of the weight of years which he carries, and there could be no doubt about his active interest in recent developments in his specialty. He said that when he compared the present condition of orthopedic surgery with that which existed fifty years ago it made him feel that he had not lived in vain. Fifty-one years ago, when he was a medical student, he ignorantly opened an abscess due to disease of the knee-joint. He was sharply taken to task by his preceptor, and the death of his patient was confidently predicted. Much to his surprise and satisfaction recovery and a useful joint followed, and his faith in professional wisdom was permanently shaken. He believed that the good result obtained then, and in many other cases afterwards treated by him, was due to the fact that in using balsam of Peru freely he had practiced antiseptic surgery without knowing it.

Dr. Gibney drew attention to the fact that the history of cases coming to him and to other orthopedic surgeons showed that many practitioners were not sufficiently careful in examining the joints in the early stage of the disease. The child saved the joint by limping and stiffness for some time before the real difficulty was suspected. Joints should be examined bare of clothing, and corresponding joints on both sides of the body should be compared. Some modification or limitation in function would then be discovered, or possibly points of tenderness, a change in contour, atrophy of the calf muscles, or increase in the knee measurement, or in the heat of one knee, would be noticed. The failure to make such an examination resulted in neglect of treatment and the advance of the disease to the stage of deformity or of abscesses. The plan of treatment preferred by him was that by immobilization until the symptoms had subsided. He quoted statistics of a large number of cases which had been treated at the Hospital for Ruptured and Crippled, which seemed to prove that the best results were obtained from this plan. Relapses had been very infrequent, and only twelve per cent. of the cases had required support during the rest of life. He was opposed to excision during early life, and exhibited two cases in which marked deformity and shortening had resulted from the operation. Several varieties of apparatus were shown, among which the Thomas splint was the type of that used by him. It consisted of a posterior leather trough, which was fastened on by a bandage.

Dr. Sayre believed in the method of treatment advocated, but would add some means of producing traction to the Thomas splint. This was necessary to protect the joint from the persistent traumatism produced by the reflex muscular contraction by which nature sought to immobilize the joint.

Dr. McBurney said that though apparatus might be necessary in the early stages, the surgeon in the meantime watching the progress of the case, a large proportion reached a state which required the use of the knife and the saw. All sections should be made above and below the epiphyseal lines, as otherwise the growth of the limb was interfered with.

### DIGITALIS IN PNEUMONIA.

The propriety of administering digitalis in pneumonia and in pulmonary œdema is a question that has attracted considerable attention ever since Dr. A. H. Smith published a paper on the subject, which was referred to in one of my letters several months ago. Dr. Smith claimed that as the strain was on the right heart rather than on the left, the indication was to dilate the blood vessels and empty the veins into the arteries. The action of digitalis was to contract the vessels and to empty the arteries into the veins; it was, therefore, contraindicated. A number of observers have objected to Dr. Smith's sweeping denunciation of the remedy, on the ground that clinical experience was favorable to its use. Amongst those who make this claim is Dr. Jacobi. During the recent discussion on pulmonary œdema at the County Society, he stated that in the acute forms of the disease, where active interference was urgently demanded, he was accustomed to give from one to four doses of digitalis. Ten grains of the powder, or the same number of minims of a fluid extract, was the quantity at each dose. Cathartics, pilocarpine, hot packs, and hot-air baths were other measures which might be indicated in special cases. Caffeine was also a remedy of great value.

Dr. Mary Putnam Jacobi expressed a similar opinion in regard to digitalis. At the meeting of the section on Pædiatrics held last evening, she reported a very severe case of infantile pneumonia, in a child nineteen months old, in which there was consolidation of the lower portion of the left lung and of the middle portion of the right, with a pleuritic effusion on the left side. The temperature reached 106° and the pulse 150, with the respiration at 72. The treatment consisted in the administration of diffusible stimulants, with the employment of cold-packs, and the inhalation of oxygen. The child recovered, but eventually an operation for empyema was required, which resulted in a sinus which was a long time in closing. During the discussion drawn forth by the relation of this history, Dr. Koplik said that a portion of rib should be resected whenever an opening was made into the chest cavity for empyema. This facilitated drainage and encouraged the rapid closure of the cavity.

Dr. McBurney approved of Dr. Koplik's view, and added that if the resection was subperiosteal no permanent injury was inflicted.

### PERITYPHLITIS.

We have not yet heard the last of this subject. The question which seems to be most prominent at present is not: "Shall an operation be performed?" that seems to be settled, but "When is the proper time for its performance?" What is meant by "early," and what by "late?" These were the questions asked very pertinently by Dr. Jacobi at the last meeting of the County Society, when he narrated the histories of two cases illustrating the difficulty in deciding these points.

In replying to the questions, Dr. McBurney stated that most "early" operations were performed in the first four days, most "late" ones after the first ten days. The question was not one of time, however, but of the condition of the patient. It was not necessary to wait until pus formed. This should be anticipated. If the patient was regarded as seriously ill, and radical treatment of some kind was required, the operation offered the best prospects. Dr. McBurney discussed the subject still further at the last meeting of the section on Pædiatrics. He stated there that he had met physicians who claimed that appendicitis never killed, and there were a large number who believed that an operation was indicated only as a last resort. Formerly, it rested with the surgeons to show proof that the operation was a real and comparatively safe remedy; now, the position had changed, and the burden of proof rested with the physicians. In judging of the time for operating the constitutional symptoms were not as safe an indication as the local conditions. A chill did not occur, and the temperature varied from normal to 103.5°. The pulse, too, might not indicate the gravity of the case. Much more reliance was to be placed on the local signs. Increasing pain, with more and more marked tenderness, more easily detected at each examination, were signs not to be disregarded. The most important point to be gained at present, was an acknowledgment that there was a remedy. If a curative remedy, such as quinine in malaria, or mercury in syphilis, had been discovered, it should be accepted. In the experience of all the New York hospitals, the results from operation for appendicitis were so superior to those obtained by any other plan of treatment, as to render comparison ridiculous. The mortality among those treated by other plans of treatment was over twenty or twenty-five per cent., not to speak of the cases in which a diagnosis was not made, of which, judging from his experience, there must be a large number. The mortality after operation was very much less than ten per cent. He had only seen one death. He had been unable to formulate a set of symptoms which would form a guide to others in determining the time to operate. He did not regard this as extremely important. Every physician was able to decide when his patient was so sick as to demand radical treatment. He showed this by making repeated visits or calling a consultation. This was the time to operate. Also, whenever an exact diagnosis could be clearly made, the remedy should be applied. Dr. L. A. Stimson remarked that if an operation was to be successful, it must be done early. The mortality after operations was only one or two per cent. In his own experience, and among his friends with whose work he was familiar, he had not known of a single death, except when general peritonitis was present. Whenever the diagnosis was made, the operation should be performed.

### DIPHTHERIA.

At the last meeting of the section on Pædiatrics, Dr. J. Lewis Smith gave a short report of the year's contribution to the knowledge of diphtheria. He made the extraordinary and startling statement that the mortality of this disease was 140 to 100,000 of the population in America, while in England it was 49 o 100,000. He said that continued experience with the following antiseptic vapor seemed to indicate that it exercised some influence in preventing the spread of the disease. It enabled physicians to visit other patients with impunity. It was even claimed by some that its use prevented diphtheritic paralysis. The formula was as follows:

℞. Acid. carbolici,
Ol. eucalypti, aa ℥i.
Spt. terebinthinæ, ℥viii.

M. Sig.—Two tablespoonfuls in a quart of water to be kept simmering on a stove in the sick room.

The chloride of iron in some form, which had been first used in diphtheria in 1859, was still in general use in France, Germany, Great Britain and America. Benzoate of sodium was also used in France in large doses. Salicylic acid and resorcin were used by some, but it had been shown that their germicidal power was very faint. He thought that prompt prophylactic measures and local treatments were the only measures on which any great reliance could be placed. A long list of local remedies had been introduced during the past year, most of them being valueless. The use of peroxide of hydrogen had extended. It was scarcely inferior to bichloride of mercury as a germicide and was not poisonous nor irritating to the mucous membrane. It could be used as a spray pure, or for young children diluted with equal parts or twice the quantity of water. When used in the nose, it should be diluted three or four time. Acetic acid as a spray or gargle has been recommended by some. A new method of local treatment has been introduced by Dr. Seibert of this city. It consisted in the injection of peroxide of hydrogen into the deeper tissues of the part affected by means of a needle with several prongs attached to a hypodermic syringe.

### LA GRIPPE.

The mysterious influenza (influence) is again at work among us. During the past two months it has helped to swell the death rate considerably, and has contributed largely to the mortality of other epidemic and systemic diseases. The same general symptoms have been noted as in former visitations, but the three varieties, noted elsewhere, have made themselves more apparent. In some cases nervous symptoms only are present, such as headache, pains in the back and limbs and neuralgia. Some of these cases are afebrile, the most pronounced symptoms being mental depression, sometimes with suicidal tendencies. The catarrhal form is that most commonly seen. In this the disease seems to manifest itself principally by an intense inflammation of the nasal passages, the pharynx and air passages. Several cases of the so-called gastric variety have also been observed here. In this there are, besides the general constitutional symptoms common to all varieties, pronounced gastro-enteric symptoms, such as pain, vomiting and diarrhœa. In a recent paper read at the Clinical Society of London, the view was taken that influenza was an infectious nervous fever due to a special poison circulating in the blood and causing congestion of the medulla oblongata. The different varieties which had been noticed depended on the part of the bulb principally affected. Thus in the nervous form the thermolytic, cardiac and other centres were especially congested. In the catarrhal variety, the congestion was largely confined to the nervous mechanism formed by the neuclei of the fifth and the vago-accessory nerves, while in the gastric form the vomiting centre was congested and the shock was transmitted to the splanchnic nerves, which anastomosed with the pneumogastric in the coliac plexus. A similarity between the poison of influenza and that of syphilis was noticed, in that both showed a tendency to attack all parts of the nervous system after the acute symptoms had subsided. The poison of influenza surpassed that of syphilis in its virulence and rapidity of action.

The question of the contagiousness of influenza is still unsettled. The general opinion among observers seem to be that it is not contracted by one patient from another.

151 *East* 50*th St.* Wm. L. Russell.

---

Treatment of Broncho-Pneumonia in Children.—Simon recommends the following prescription in the early stages of this condition:

℞. Acetate of ammonium, 7 grains.
Tincture of aconite, 15 drops.
Codeine, 2 grains.
Simple syrup, ½ ounce.
Water enough to make 3 ounces.

A teaspoonful of this may be given every hour until five or six doses are taken. At the beginning of an attack a mustard plaster or other form of counter irritation should be applied over the thorax, and after it has acted for one or two hours should be followed by an emollient poultice. It is also well to allow steam from boiling water to pass into the air of the sick-room.—*L' Union Medicale.*

